# Response to Comment on Thigpen D. et al. The Role of Ultrasound in Screening Dense Breasts—A Review of the Literature and Practical Solutions for Implementation. Diagnostics 2018, 8, 20

**DOI:** 10.3390/diagnostics8020038

**Published:** 2018-05-24

**Authors:** Denise Thigpen, Amanda Kappler, Rachel Brem

**Affiliations:** Department of Radiology, The George Washington University, Washington, DC 20037, USA; akappler@mfa.gwu.edu (A.K.); rbrem@mfa.gwu.edu (R.B.)

Thank you for your thoughtful comments regarding breast density legislation. We welcome the opportunity for deeper engagement on this important topic. There is great variation in the language used in individual state laws informing women of breast density and we regret that this important discussion was beyond the scope of our manuscript. As discussed in the comment, some states only inform women of the concept of dense breast tissue and do not inform the woman of her individual breast density. This can cause confusion for a woman receiving the letter about her own personal status and what her next steps should be. There is a need for more universal language disclosing the woman’s personal density as well as the benefit of additional screening. Many radiologists are familiar with the calls from worried patients who have just received their letter in the mail regarding breast density. This is an opportunity to provide patient-centered, value-added care. Our expertise as radiologists provide us with an opportunity to inform, educate and advise the patient on the issue of breast density. Radiologists are the best equipped to discuss the potential benefits (such as increased cancer detection) and potential disadvantages (such as false positives) that are inherent to adjunct screening modalities, such as MRI and Ultrasound. As the legislative landscape continues to evolve, radiologists should strive to engage with their patients in order to lead the way forward in patient-centered care.

